# An Eventful Week

**Published:** 1920-11-20

**Authors:** 


### THE MULTITUDE OF WORDS.

### An Eventful Week.

"Zounds! I was never so bethump'd with words
Since I first called my brother's father dad."

Hospitals and nurses have certainly had their innings last week, and I hope everybody is quite content. Who was the oracle who remarked that when one is contented there is no more to be desired, and when there is no more to be desired there is an end of it?

My own feeling after reading the multitude of words is that I want to sigh. The report of the debate in Parliament, and the verbatim report in The Hospital of the College of Nursing meeting have jointly and severally produced this effect. I am sure very few nurses read the report of the debate in Parliament on the Ministry of Health Bill. It occupied two days, and only a small fraction of it appeared in the morning papers. There is, however, more to be gathered from the single fact that of the seven hundred Members of Parliament who were supposed to be taking an interest in the Bill, only 229 actually voted, there being 177 ayes and 52 noes. This is bad enough, but Sir Henry Craik, an extremely intelligent gentleman, in discussing the Bill, said that although he had spoken to twenty-five or thirty members about it, not more than two or three had even looked at the Bill, and that if we knew all the facts he was not sure that the Cabinet as a whole knows very much more about it than honourable members. Not more than one or two of the right hon. gentleman's colleagues, Sir Henry concluded, would be able to stand anything but a very cursory examination upon the principles of the Bill.

### A Missing Word.

This is the Bill that has been before Parliament when the immediate and ultimate fate of voluntary hospitals has been under discussion, and for which obscure people like nurses expect so much light and leading. I doubt, indeed, if the word "nurse" was once mentioned throughout. I do not remember meeting with it.

I am trying to explain why I had to sigh.

Then I turn to the verbatim report of the College "extraordinary" meeting, and when I saw that Lord Knutsford rose to speak I was full of expectation. Lord Knutsford had announced his intention of closing the "London" on New Year's Day, and presumably if he does so nurses and patients will be literally in the street. I do not know what the nurses comprising the "extraordinary" meeting of the College expected to hear from Lord Knutsford in the circumstances. I should think that an important pronouncement of some sort was hoped for. To my mind, Lord Knutsford's speech, though doubtless well meant, is bound to be most damaging to the College. The pith of his speech is that the College is too young to be of any use. And he admits that many critics do not think that such as he should be on the Council at all. I wonder if he thinks that this speech will cause the critics to change their view. It will certainly add to their number. It is a monstrous affront to 20,000 full-grown trained and professional women to tell them that it is impossible at this hour for the College to do anything. The doctrine is quite enough to shatter the fabric to splinters.

But, in truth, it is very difficult to allocate blame in a case of this kind. Lord Knutsford is on the Council as the result of a free election, and, although his lordship admits that nurses cannot know whom to vote for, it is apparent that there is a most deplorable apathy in the ranks. Sir Arthur Stanley says that electors put their voting-papers in the wastepaper basket, and Lord Knutsford suggests that they lit their cigarettes with them.

### Too Young to be Told.

All this, while the fate of the whole nursing profession is in grave and imminent danger of disaster. It reminds me of reports which I used to read during the war of the Russian Armies holding committee meetings while they were being encircled by the enemy. I suppose the nurses are too young to be told! Are we also too young to be told how a statement on one page of *The Times* that the "London" will close on January 1 is to be reconciled with another on a different page of the same issue, and, coming from the same authority, announcing a new salary scale for "London" nurses to commence from the date of its closing? One does not expect too much from a Parliamentary debate. There is always the unknown quantity of politics, party manœuvring, and jealousies. But in a general meeting between the College Council, and such of its members as were able to attend, one expects in a crisis like the present a heart-to-heart discussion of vital matters, instead of afternoon-tea persiflage.

I observe that the Chairman at this meeting appealed for suggestions as to what the College Council might do, and I do not see any reply. To me this is not conclusive evidence that the nurse's mind is a blank. It is not an uncommon thing in the Army for an officer to ask his men on parade—"any complaints?"—and, although no answer is forthcoming, for the officer to hear from another source that the ranks are seething with discontent. I am doubtful if the College surface and its undercurrents correspond, but I want to be sure.

I shall, therefore, esteem it a favour if some nurses will communicate with me on this subject. Is there, in their opinion, anything that they think the College Council ought to do? Is there a consciousness, felt and admitted, that they and their magnificent institution is a baby made to do ought save to babble blessings for being wheeled about by eminent councillors? I desire to know this for my personal interest, but in a far greater degree for the interest of the College. Address "Ierne," Hospital Offices. And, in conclusion, may I quote St. Paul: "Let no man despise thy youth!"

IERNE.

